# Concurrent features of sarcoidosis and hypersensitivity pneumonitis in two patients exposed to fungal antigens

**DOI:** 10.1186/s12890-023-02642-x

**Published:** 2023-11-04

**Authors:** Almerico Marruchella, Paola Faverio, Fabrizio Luppi

**Affiliations:** 1 Respiratory Disease, Fondazione IRCCS “San Gerardo dei Tintori”, Monza, Italy; 2 Department of Medicine and Surgery, University of Milan-Bicocca, Milano, Italy

**Keywords:** Hypersensitivity pneumonitis, Sarcoidosis, Granulomatous inflammation, Diagnosis

## Abstract

**Background:**

Sarcoidosis and hypersensitivity pneumonitis (HP) are two distinct clinical entities that share granulomatous inflammation, although each of them has specific clinical, radiologic and pathologic profiles. Coexistence of the two diseases have been described, suggesting, at least in some cases, a common biologic background.

**Case presentation:**

We describe two patients showing the concurrent diagnosis of sarcoidosis and hypersensitivity pneumonitis. Case 1: a 51-year old never smoker man had a history of occupational exposure, episodes of acute exacerbations and positive serum precipitins to* Penicillium spp* suggestive of HP, while the positivity of serum angiotensin converting enzyme (ACE) favored sarcoidosis. Case 2: a 42-year old non-smoker woman with occasional finding of enlarged mediastinal lymph nodes had a history of domestic exposure to molds and positive serum precipitins to *Aspergillus spp* suggestive of HP.

In both cases high resolution computed tomography (HRCT) together with broncoscopy findings allowed to maintain both the diagnoses: HRCT showed both enlarged hilar/mediastinal limph nodes and intersitial lung involvement typical of HP; bronchoalveolar lavage presented marked lymphocytosis and granulomatous nodal lesions were observed at transbronchial needle aspiration.

**Conclusions:**

Sarcoidosis and HP share some clinical findings and the differential diagnosis may be difficult. Our cases suggest that a common trait may be responsible for the concurrent diagnosis of sarcoidosis and hypersensitivity pneumonitis in the same patient.

## Background

Sarcoidosis and hypersensitivity pneumonitis (HP) are two distinct clinical entities that share granulomatous inflammation, although each of them has specific clinical, radiologic and pathologic profiles.

Sarcoidosis is a systemic disease of unknown etiology [1], although there is evidence that infectious agents such as bacteria, particularly Propionibacterium acnes, both tuberculous and nontuberculous mycobacteria and fungi have been suspected as being a possible cause of sarcoidosis. Nevertheless, non-infectious agents represent also risk factors for the occurrence of sarcoidosis with numerous environmental agents, including working in various occupations, such as exposure to different substances, and dwelling in particular environments [2]. Whereas, HP is an immune-mediated lung disease caused by inhalation of various antigenic substances in predisposed individuals. In HP the exposure in many instances is related to workplace and the disease has legal implications mainly related to compensation for affected workers and a responsibility for the employer to control the exposures [3].

In pulmonary sarcoidosis histology typically reveals well-formed non caseating epithelioid granulomas with perilymphatic distribution and bronchoalveolar lavage (BAL) shows a lymphocytosis (usually moderate, in the range 30–40%) sustained by Th1 CD4 + T-lymphocytes). In a small proportion of cases the predominant cells are CD8 + with a Tc1 cytokine profile. High resolution computed tomography (HRCT) usually shows a micro-nodular peri-lymphatic pattern and bilateral hilar and mediastinal adenopathies. Diagnosis relies upon a compatible clinical picture, histologic demonstration of non-caseating granulomas, and exclusion of other granulomatous diseases [1].

HP is characterized by a marked mononuclear infiltration of pulmonary interstitium, more evident in the peribronchiolar/centrilobular compartment; loose granulomatous structures or isolated giant cells can be seen. The distinctive feature of BAL is a marked lymphocytosis, often > 80%, with a variable CD4+/CD8 + ratio. In non-fibrotic HP exposed individuals develop a flu-like illness 4–6 h after exposure, resolving spontaneously or with corticosteroids. In fibrotic forms the clinical presentation is more insidious and non-specific (mainly dyspnea on effort and cough). In the non-fibrotic form HRCT may be characterized by ill-defined centrilobular ground-glass nodules; in fibrotic HP signs of fibrosis are present, sometimes mimicking a UIP pattern. Recently, international guidelines have been published, using a systematic approach to the diagnosis of HP [4].

We report two cases with concomitant clinical, radiological and pathological features of sarcoidosis and HP, suggesting a common biologic background, at least in some cases.

## Cases presentation

### Case 1

**Fig. 1:**
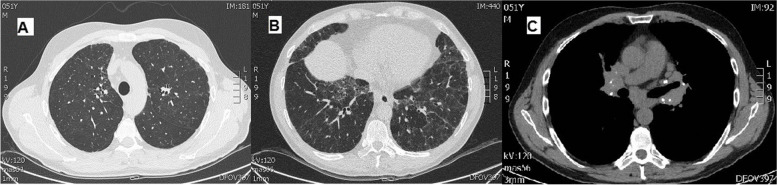
Chest HRCT showing ill-defined centrilobular opacities in the upper lobes (**A**); normal density areas, extensive ground glass attenuation and lobular areas of air-trapping (“head cheese pattern”) with reticular zones of fibrosis in the lower lobes (**B**). Mediastinal window shows hilar and mediastinal lymph nodes enlargement with small calcifications (**C**)

**Fig. 2:**
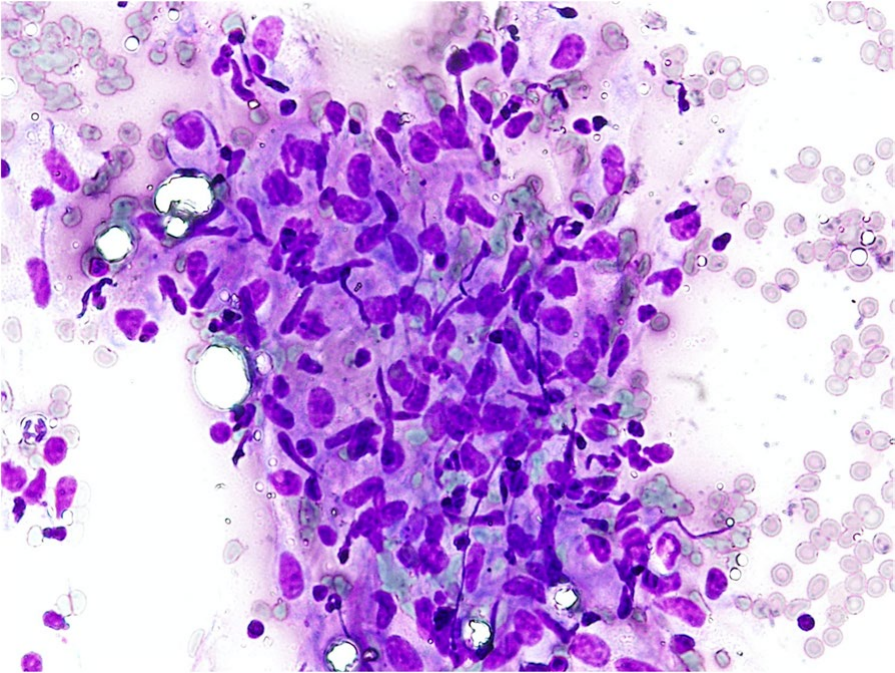
Transbronchial needle aspirate. Microgranulomatous aggregate of epithelioid histiocytes; absence of necrosis. Hemacolor®, 200x

A 51-year old never smoker man sought medical attention because of progressive exercise-related breathlessness. His medical history was unremarkable, with the exception of arterial hypertension, treated with olmesartan. He had been working since the age of 25 years in a printing works company. His family had a factory involved in the production and seasoning of sausages and cheeses. Occasionally he had been engaged in such activities, especially in the handling and brushing of sausages and cheeses. He had experienced many times an acute illness a few hours after exposure, consisting of malaise, fever, chills, dyspnea and myalgias and lasting about two days; in some occasions he had successfully taken corticosteroids to relieve symptoms. Pulmonary function tests (PFTs) were normal: Forced Vital Capacity (FVC) 4.11 L (90% of predicted value), Forced Expiratory Volume in the 1st second (FEV1) 3.17 L (86%), FEV1/FVC 77%, and Total Lung Capacity (TLC) 5.70 L (81%) with moderate reduction of Diffusing Capacity for Carbon monoxide (DLCO single breath 42%). Arterial Blood gases at rest were in the normal range. Six minute walking test (6MWD) revealed a normal distance walked (660 m) and a severe desaturation with nadir oxygen saturation of 80%. Chest x-ray and high resolution computed tomography (HRCT) were remarkable for partially calcified hilar and mediastinal lymph nodes and a diffuse infiltrative lung disease with areas of ground glass density admixed to lobular-shaped zones of hypo-attenuation, ill defined centrilobular nodules and signs of fibrosis (Fig. 1). A bronchoscopy was performed with bronchoalveolar lavage (BAL) and was negative for bacteria, mycobacteria and fungi. Cell count showed a marked lymphocytosis (86%) with a CD4+/CD8 + ratio 2.4. Laboratory tests were unremarkable with the exception of an elevated angiotensin converting enzyme (ACE, twice the normal value). He was then referred to our centre for consultation. Autoimmune laboratory tests, including Anti-nuclear antibodies (ANA), Extractable nuclear antigen (ENA), rheumatoid factor (RF) and anti-cyclic citrullinated peptides (CCP) antibodies were negative; serum precipitins revealed a strong sensitization to *Penicillium spp*. A new bronchoscopy with transbronchial needle aspiration (TBNA) of mediastinal lymph nodes disclosed a non-necrotizing granulomatous pattern with numerous aggregates of epithelioid histiocytes (Fig. 2). Transbronchial lung biopsies (TBLB) included alveolar tissue with chronic inflammatory septal infiltrate and aggregates of fibrinoid material; granulomatous structures were not found.

Taken together, many features were consistent with a diagnosis of HP: significant history of exposure, consistent serum precipitin test, various episodes attributable to acute HP, marked BAL lymphocytosis, HRCT pulmonary findings. However, the prominent hilar and mediastinal lymph nodes, along with the cytological TBNA results, strongly supported a concurrent diagnosis of sarcoidosis.

The patient was advised to avoid any future exposure and corticosteroid therapy was started. At two-years follow-up there was a functional improvements: FVC 4.51 L (97%), FEV1 3.85 L (104%), FEV1/FVC 83%, TLC 6.87 L (95%), DLCO 42%; DLCO/VA 49%.

### Case 2

A 42-year old non-smoker woman was occasionally found as having mediastinal lymph node enlargement on chest CT; pulmonary abnormalities were not found. She reported hypertension and prior left facial nerve palsy. Lymphocytosis was found at BAL (39% lymphocytes with CD4/CD8 ratio 3.5). Malignant cells and infectious agents were absent. Mediastinal sarcoidosis was diagnosed and no therapy was prescribed. Eighteen months later the patient developed progressive exertional dyspnea and a restrictive ventilatory defect with severe DLCO impairment were documented at PFTs: FVC 1.69 L (44%), FEV1 1.45 L (45%), FEV1/FVC 86%, TLC 3.19 L (55%), DLCO 33%. Prednisone was started (50 mg per day for 4 weeks, followed by slow tapering to 5 mg/die maintenance dose) and a slight functional improvement was obtained (FVC 2.05 L, 54%). Three years later, because of worsening dyspnea she attended the outpatient clinic of our hospital. At chest CT ill-defined areas of ground glass attenuation were observed as well as multiple enlarged hilar and mediastinal lymph nodes. A worsening of the known restrictive ventilatory disorder became obvious on PFTs (FVC 1.59 L, 42% of normal predicted value). BAL lymphocytes were 61% with a CD4/CD8 ratio 3.75. TBNA of subcarinal lymph nodes disclosed numerous small lymphocytes and microgranulomatous aggregates of epithelioid histiocytes. TBLB included fragments of lung parenchyma with a mononuclear septal infiltrate, alveolar foamy macrophages and a multinucleated giant cell. A careful review of medical history revealed a previously unreported domestic exposure to molds. Serum precipitins displayed reactivity to *Aspergillus spp* antigens.

After multidisciplinary discussion the case was labelled as simultaneous occurrence of HP and mediastinal sarcoidosis. Prednisone daily dose was raised to 12.5 mg per day; a clinical and functional improvement (FVC 1.9 L, 50%) was obtained.

We added in Table 1 points in favor of both Sarcoidosis and HP for both the cases.

**Table 1:** Points in favor of both Sarcoidosis and Hypersensitivity pneumonitis for both the cases

Features favoring Hypersensitivity pneumonitis		
	Case 1	Case 2
History of exposure	+	+
Episodes of acute HP	+	-
Serum precipitins	+	+
HRCT findings	+	+
Histopathological findings (TBLB)	-	+
BAL (Ly > 50%)	+	+
Features favoring sarcoidosis		
	Case 1	Case 2
Hilar/mediastinal adenopathies	+	+
Granulomatous nodal lesions (TBNA)	+	+
A.C.E.	+	+

## Discussion and conclusions

Sarcoidosis and HP share some clinical findings and the differential diagnosis may be difficult, especially in patients without a history of professional or environmental exposure. In 1981 *Konig and colleagues* revised a series of 46 cases of sarcoidosis: 14 of them had a history of significant exposure and inhalation challenge favoured a diagnosis of HP [5].

In our cases the association between sarcoidosis and HP is supported by clinical, laboratory, imaging and pathological findings. In particular, both had voluminous bilateral hilar and mediastinal lymph nodes and non necrotizing granulomatous aggregates of epithelioid cells: these findings are typical of sarcoidosis. History, lung imaging and laboratory tests are all consistent with HP. The list of antigens potentially causing HP in predisposed individuals is broad and continuously growing [2]. In both cases the history and precipitins test are convergent in identifying the inciting agent of HP in a fungal antigen (*Penicillium spp* and *Aspergillus spp*, respectively). On the other hand, although the pathogenesis of sarcoidosis has been relatively elucidated, the trigger remains elusive. Infectious organisms (e.g. mycobacteria, *Propionibacterium spp*) or environmental exposures are possibly involved in some cases but a definitive evidence is lacking [5].

In HP, epithelioid histiocytes predominate only focally, usually around the airways, resulting in a subtle granulomatous appearance. Well-formed (sarcoid-like) granulomas are rare. Isolated multinucleated giant cells are common and, in a minority of cases, represent a striking feature. In contrast, discrete well-formed non-necrotising granulomas are the histological hallmark of sarcoidosis. Granulomas comprise a compact circumscribed cluster of epithelioid and multinucleated histiocytes with minimal or no central necrosis. They are often associated with a peripheral infiltrate of lymphocytes and a distinctive pattern of lamellar fibrosis [6].

Finally, both sarcoidosis [7] and HP [8] have been linked to a predisposing genetic background, particularly to polymorphisms of major histocompatibility genes (MHC). Although a fortuitous association cannot be ruled out, our cases suggest that in some patients a common genetic trait may be responsible for a strong immune response to environmental antigens leading to clinico-pathological findings consistent with both sarcoidosis and HP.

## Data Availability

Not applicable.

## Abbreviations

HP: Hypersensitivity pneumonitis
BAL: Bronchoalveolar lavage
HRCT: High resolution computed tomography
FVC) 4: Forced Vital Capacity
FEV1: Forced Expiratory Volume in the 1st second
TLC: Total Lung Capacity
DLCO: Diffusing Capacity for Carbon monoxide
6MWD: Six minute walking test
ACE: Angiotensin converting enzyme
ANA: Anti-nuclear antibodies
ENA: Extractable nuclear antigen
RF: Rheumatoid factor
CCP: Anti-cyclic citrullinated peptides
TBNA: Transbronchial needle aspiration
TBLB: Transbronchial lung biopsies
